# Nightly Sleep Duration and Symptom Burden Over 1 Month Following Pediatric Concussion

**DOI:** 10.1001/jamanetworkopen.2025.16333

**Published:** 2025-06-18

**Authors:** Lauren Butterfield, Roger Zemek, Michael M. Borghese, Vid Bijelic, Nick Barrowman, Veronik Sicard, Nicholas Kuzik, Mark S. Tremblay, Keith Owen Yeates, Andrée-Anne Ledoux

**Affiliations:** 1 Children’s Hospital of Eastern Ontario Research Institute, Ottawa, Canada; 2 Department of Neuroscience, Carleton University, Ottawa, Canada; 3 Department of Pediatrics, University of Ottawa, Ottawa, Canada; 4 Environmental Health Science and Research Bureau, Health Canada, Ottawa, Canada; 5 Department of Psychology, University of Calgary, Calgary, Canada; 6 Department of Cellular and Molecular Medicine, University of Ottawa, Ottawa, Canada

## Abstract

**Importance:**

Approximately half of all youths with concussions report sleep disturbance, including short and long sleep durations, in the first week of recovery. Limited longitudinal research exists regarding the association between sleep duration and symptom burden during acute pediatric concussion recovery.

**Objective:**

To investigate the association between mean nightly sleep duration over 1 week (days 1 to 7) and 2 weeks (days 1 to 14) postinjury and subsequent symptom burden at 1, 2, and 4 weeks following pediatric concussion.

**Design, Setting, and Participants:**

This cohort study used data from a randomized clinical trial conducted in 3 emergency pediatric departments in Ontario, Canada, from March 2017 to December 2019 (with secondary analysis conducted between September 2022 and September 2024). Eligible participants were between ages 10 years and 18 years who received treatment for a concussion within 48 hours.

**Exposure:**

Sleep postinjury was measured with waist-worn accelerometers 24 hours per day for 2 weeks and daily sleep logs.

**Main Outcomes and Measures:**

Symptom burden was measured with the Health and Behavior Inventory at 1 week, 2 weeks, and 4 weeks postconcussion. A nonlinear mixed-effects model was applied that estimated symptom burden at 1, 2, and 4 weeks from mean sleep duration over days 1 to 7 and days 1 to 14. Logistic regressions were performed to assess the odds of being reliably symptomatic at 2 and 4 weeks by mean sleep duration. Conservative (*z* ≥ 1.65) and liberal (*z* ≥ 1.28) definitions of reliable change in symptoms were evaluated. All models compared 10th vs 50th, 25th vs 75th, and 50th vs 90th percentile contrasts, and were adjusted for prognostic pre-injury demographic and baseline injury variables.

**Results:**

A total of 291 participants (median [IQR] age, 13.2 [11.6-14.9] years; 128 female [44.0%]) were included in the main analysis. Mean nightly sleep duration beyond 9.5 hours in the first week postconcussion was associated with higher symptom burden at 1 week (75th percentile [10.5 h] vs 25th percentile [9.5 h]: estimate, 1.3 [95% CI, 0.25-2.28]; 90th percentile [11.3 h] vs 50th percentile [10.0 h]: estimate, 2.9 [95% CI, 1.22-4.69]). Mean sleep duration beyond 9.9 hours in the first 2 weeks postconcussion was associated with higher symptom burden at 2 weeks (90th percentile [10.9 h] vs 50th percentile [9.9 h]: estimate, 2.2 [95% CI, 0.85-3.47]) and 4 weeks (estimate, 2.2 [95% CI, 0.85-3.47]), and increased odds of persisting symptoms at 4 weeks (conservative: odds ratio [OR], 1.73 [95% CI, 0.91-3.26]; liberal: OR, 1.93 [95% CI, 1.07-3.47]).

**Conclusions and Relevance:**

In this observational study of adolescents, average nightly sleep durations beyond 9.9 hours over the first 2 weeks of concussion recovery were associated with high symptom burden and persistent symptoms. Clinicians should monitor youths’ sleep after concussions.

## Key Points

**Question:**

Is there an association between average nightly sleep duration and symptom burden within 1 month of pediatric concussion?

**Findings:**

This cohort study of 291 Canadian youths treated for concussions found that average nightly sleep durations beyond 9.5 hours over the first week and 9.9 hours over the first 2 weeks of concussion recovery was associated with higher symptom burden at 1, 2, and 4 weeks postconcussion, and increased odds of persisting symptoms at 4 weeks.

**Meaning:**

These results suggest that nightly sleep duration was associated with increased symptom burden over the first month of concussion recovery; therefore, clinicians should monitor youth’s sleep after concussion.

## Teaser

This cohort study of Canadian youths treated for concussions examined the association of average nightly sleep duration with symptom burden over 4 weeks.

## Introduction

Approximately 30% to 35% of children will have persisting symptoms after a concussion (PSAC) beyond 1 month postinjury, including somatic, emotional, sleep, and/or cognitive abnormalities.^1,2,3^ PSAC may impair daily activities, quality of life,^4^ and mental health,^5^ highlighting the pressing need for further research to better understand these symptoms, their correlates, antecedents, and determinants.

Healthy sleep in youth is associated with reduced stress and improved psychological outcomes, emotional regulation, academic performance, and health behaviors.^6,7,8,9^ Disruptions to healthy sleep patterns are associated with poor mental health, mood problems, and behavioral and functional impairments.^7,10,11,12^ Interestingly, healthy adolescents with reduced sleep durations experience concussion symptoms without having suffered any brain injuries.^13^

Pre- and postconcussion sleep disturbances are thought to be associated with higher concussion symptoms and prolonged recovery.^14,15,16^ Approximately 51% of children experience sleep disturbances in the first week of acute pediatric concussion, with the most common being drowsiness and increased sleep.^17^ In previous pediatric concussion studies, poor sleep quality and short sleep duration have been associated with anxiety and depression,^18,19,20^ higher symptom burden,^18,19,21,22,23^ prolonged recovery time,^18,19^ and delayed return to school.^14^ Others have found no or mixed associations between sleep duration and symptom burden, PSAC or recovery.^24,25,26,27,28,29^ However, these studies are limited by small sample sizes,^14,23,26,29,30,31^ the inclusion of sports-related concussion only,^19,22,23,25,27,30^ or the use of self-reported sleep duration.^18,21,22,27^

The lack of rigorous prospective studies on the associations between sleep duration and symptom burden that include objective measures has made it challenging to evaluate and establish their relationship following pediatric concussion. Wearable technology, such as accelerometers or actigraphy, can be used to monitor youths’ movements and estimate sleep measures^32,33,34,35^; however, limited studies^14,20,23,24,25,26,28,29,30,31^ have used this technology to evaluate the relationship between sleep duration and pediatric concussion recovery. This study had 2 goals: (1) To investigate the association between sleep duration and subsequent symptom burden at 1, 2, and 4 weeks postinjury; and (2) to examine the association between sleep duration and reliable change in symptoms at 2 and 4 weeks. We hypothesized that longer sleep duration during the first 2 weeks after concussion would be associated with reduced symptom burden at 1, 2, and 4 weeks postconcussion and lower odds of being reliably symptomatic at 2 and 4 weeks.

## Methods

### Study Design and Setting

This planned secondary analysis, using a cohort study design, was conducted using data from the Pediatric Concussion Assessment of Rest and Exertion study (PedCARE) (NCT02893969),^36^ a multicenter clinical trial. The PedCARE study was conducted between March 2017 and December 2019 in 3 Canadian Pediatric emergency departments (ED).^37^ The current analysis was performed between September 2022 and September 2024. Ethical approval for the PedCARE study was obtained from the research ethics board of all 3 institutions. Written informed assent or consent was obtained from all participants, with parental consent also provided. This study adheres to the Strengthening the Reporting of Observational Studies in Epidemiology (STROBE) reporting guidelines for observational studies.

### Population

This study included youths (ages 10 up to 18 years) presenting to the ED within 48 hours of a concussion. Concussions were defined according to the Zurich/Berlin consensus statements for sport-related concussion.^38,39^ To confirm concussions in participants, an adapted version of the Centers for Disease Control and Prevention (CDC) tiered framework was utilized^40^ and outlined elsewhere.^41^ Exclusion criteria included a Glasgow Coma Scale score of 13 or below; abnormalities on brain imaging, neurosurgical intervention, intubation, or admission to the intensive care unit; multisystem injury requiring hospitalization; severe preexisting neurological developmental delay resulting in communication challenges; intoxication; absence of trauma history as the primary event; previous enrollment in the study; insurmountable language barriers; and an inability to complete follow-up assessments.

### Study Protocol

The study protocol and primary analyses are published.^36,37^ Upon recruitment, participants provided information on demographics, injury, personal health status, and mental health history. Additionally, balance was evaluated using the Balance Error Scoring System^42^ to compute the Predicting Persistent Postconcussive Problems in Pediatrics (5P) risk score.^2^

Pre- and postinjury symptoms were measured with the Health and Behavior Inventory (HBI). The HBI is a validated and reliable questionnaire (20 items; rated 0-3; total range, 0-60) that measures cognitive (11 items; range, 0-33) and somatic (9 items; range, 0-27) symptoms.^43,44,45^ Higher scores indicate higher symptom burden. A parent-reported retrospective HBI (rHBI) was completed at baseline (ED) to obtain preinjury scores. HBI was self-reported at baseline, 1, 2, and 4 weeks postinjury via a Research Electronic Data Capture (REDCap)^46,47^ database or phone.

Participants were randomized to either the experimental (return-to-physical activity at 72 hours) or control (rest until asymptomatic) groups for the parent study^36,37^ and unrelated to the current cohort study. Each participant was provided with an accelerometer to measure daily movement behaviors. The accelerometer was used to estimate bedtimes and waketimes by monitoring activity counts 24 hours per day for 14 consecutive days, beginning at midnight following enrollment. The accelerometer was worn on a belt around the waist at the right midaxillary line, and data were collected in 1-minute epochs (excluding aquatic activities). During these 14 days, participants and/or their parent proxy recorded their bedtimes and waketimes via daily REDCap surveys or phone.

Preinjury sleep symptoms were rated using self-report preinjury Post-Concussion Symptom Inventory (PCSI)^48^ at 2 weeks postconcussion.^49^ Two PCSI items were included in the current study: preinjury tiredness and drowsiness (range, 0-2).

### Outcome Measures

The descriptive analysis included bedtime, waketime, sleep duration, and sleep midpoint. Sleep duration was calculated as the amount of time between bedtime and waketime values obtained through self-report and verified or estimated with actigraphy. Sleep midpoint was calculated as the time of day halfway through the sleep duration. These 4 sleep measures were reported for both weeknights (Sunday-to-Thursday nights) and weekend nights (Friday-to-Saturday nights).

Symptom burden was quantified as the total score, as well as cognitive and somatic symptom scores, derived from the HBI.^43,44^ Participants were grouped as reliably symptomatic or asymptomatic using reliable change *z* scores at 2 and 4 weeks postconcussion.^45^ This metric compares the parent-reported rHBI with the participant-reported HBI during the 2- and 4-week follow-up periods, using formulae derived from regression analyses conducted on youths with orthopedic injuries. Symptomatic participants were defined with the conservative reliable change *z* score of 1.65 or higher, signifying a greater-than-expected symptom increase. A liberal reliable change *z* score of 1.28 or higher was also evaluated.

#### Bed and Waketime Data Processing

An accelerometer (Philips Respironics) measured activity counts in 1-minute epochs, which yielded daily actograms for each participant using Actical version 3.10.0001 (Respironics, Inc). The actograms are activity count vs time graphs that were used to visually verify the bedtime and waketime values provided in sleep logs.^32,33,34^ In the event of missing sleep logs, bedtimes and waketimes were estimated based on Actograms in participants who wore the accelerometer overnight. These methods were performed by 2 authors (L.B. and M.M.B.) (eMethods in Supplement 1).

### Statistical Analysis

Complete sleep data in week 1 was defined as 4 or more nights of sleep data during days 1 to 7.^23,25,34,35^ Complete sleep data over weeks 1 and 2 (days 1 to 14) was defined as 4 or more nights of sleep data during days 1 to 7 and 4 or more nights of sleep data during days 8 to 14.

To assess the association between mean sleep duration and symptom burden at 1-, 2-, and 4-weeks, a nonlinear mixed-effects model was performed with HBI at 1-, 2-, and 4-weeks as primary outcomes, mean sleep duration over week 1 (days 1 to 7) and over weeks 1 and 2 (days 1 to 14) as a time-varying independent variable, and fixed effects of age, sex, randomization group, 5P risk score,^2^ preinjury mental health diagnoses (binary variable), baseline HBI, rHBI, and PCSI pre-injury tiredness and drowsiness ratings (eMethods in Supplement 1). The PCSI items were included to control for preinjury fatigue variability.

To investigate the association between mean sleep duration and odds of being reliably symptomatic at 2 and 4 weeks, multivariable logistic regression models with odds ratio were computed. The covariates were the same as the previous model.

Two sensitivity analyses were conducted with identical models to the main nonlinear mixed effects model. The first investigated the impact of self-reported and accelerometer-estimated bias by excluding participants who had less than 80% of sleep duration measures confirmed by sleep logs and overnight Actograms. A sleep duration measure was confirmed if that day included sleep log values, and the accelerometer was worn overnight. Four statistical outliers with mean sleep duration of *z* ≥ 3.29 or *z* ≤ −3.29 were excluded from the first sensitivity analysis (*z* = −4.6, 3.5, 3.7, 4.9).

The second sensitivity analysis was conducted to adjust for cumulative moderate-to-vigorous physical activity (MVPA), given that MVPA can influence concussion symptom burden.^41^ MVPA was calculated based on daily activity counts from the accelerometer, using published methods.^37,41^

Exploratory secondary analyses identical to the main analysis with the HBI cognitive and somatic subscores at 1, 2, and 4 weeks as primary outcomes were completed. The total rHBI and baseline HBI variables were replaced as fixed effects with the respective subscores.

The assumption of linearity was violated for the mean sleep duration, cumulative MVPA, and age variables. Therefore, all models included nonlinear restricted cubic splines (3 knots) on the mean sleep duration, cumulative MVPA, and age variables. As an additional response to nonlinearity, all analyses compared 10th vs 50th, 25th vs 75th, and 50th vs 90th percentiles difference contrasts. Statistical significance was defined as *P* < .05 in 2-tailed tests. The analyses were conducted in R version 4.2.1 (R Project for Statistical Computing).

## Results

Of 456 participants, 291 youths (median [IQR] age, 13.2 [11.6-14.9] years; 128 female [44.0%]) were included in the main analysis (eFigure 1 in Supplement 1). Participants slept a mean (SD) duration of 10.0 (1.7) hours per day over week 1 (days 1 to 7) and 9.8 (1.7) hours per day over weeks 1 and 2 (days 1 to 14) (Table 1). Bedtime, waketime, and sleep midpoint values are reported in Table 2. The percentiles of mean sleep duration for each analysis are presented in eTable 1 in Supplement 1. Locally estimated scatterplot smoothing curves based on total HBI vs mean sleep duration at 1, 2, and 4 weeks indicated significant nonlinearity for the main mixed-effects model (eFigure 2 in Supplement 1).

**Table 1.** Personal Characteristics and Demographics

Variable	Youths, No. (%)		
	Main analysis (N = 291)	Sensitivity analysis 1 (n = 184)	Sensitivity analysis 2 (n = 129)
Age, median (IQR), y	13.2 (11.6-14.9)	13.4 (11.6-15.0)	12.5 (11.0-14.2)
Sex			
Female	128 (44.0)	83 (45.1)	60 (46.5)
Male	163 (56.0)	101 (54.9)	69 (53.5)
Site specific sample			
Children’s Hospital of Eastern Ontario (CHEO)	202 (69.4)	133 (72.3)	97 (75.2)
The Hospital for Sick Children (SickKids)	54 (18.6)	24 (13.0)	21 (16.3)
The London Children’s Hospital	35 (12.0)	27 (14.7)	11 (8.5)
5P clinical risk score			
Low risk (0-3)	17 (5.8)	11 (6.0)	8 (6.2)
Medium risk (4-8)	219 (75.3)	138 (75.0)	102 (79.1)
High risk (9-12)	55 (18.9)	35 (19.0)	19 (14.7)
Time from head injury to triage, median (range), h^a^	3.3 (0.3-47.6)	3.9 (0.5-47.0)	3.1 (0.3-47.0)
Previous migraine history	16 (5.5)	9 (4.9)	5 (3.9)
Previous concussion history	93 (32.0)	63 (34.2)	34 (26.4)
Previous number of concussions, median (range)	1 (1-6)	1 (1-6)	1 (1-5)
Previous concussion symptom duration beyond 1 week	66 (71.0)	45 (71.4)	23 (67.6)
Diagnostic history			
Learning disability	43 (14.8)	27 (14.7)	17 (13.2)
Attention-deficit/hyperactivity disorder	43 (14.8)	30 (16.3)	19 (14.7)
Other developmental disorder	16 (5.5)	14 (7.6)	8 (6.2)
Anxiety	53 (18.2)	33 (17.9)	18 (14.0)
Depression	18 (6.2)	8 (4.3)	5 (3.9)
Sleep disorder	12 (4.1)	9 (4.9)	6 (4.7)
Other psychiatric disorder	5 (1.7)	4 (2.2)	3 (2.3)
Mechanisms of injury			
Occupant in motor vehicle collision	2 (0.7)	1 (0.5)	2 (1.5)
Motorcycle accident	0	0	0
Motorized recreation vehicle accident	0	0	0
Pedestrian struck by automobile	1 (0.3)	1 (0.5)	1 (0.8)
Bike struck by automobile	0	0	0
Bike collision or accident/fall	2 (0.7)	2 (1.1)	1 (0.8)
Fall from elevation	11 (3.8)	7 (3.8)	5 (3.9)
Fall downstairs	6 (2.1)	3 (1.6)	3 (2.3)
Fall from standing, walking, or running	39 (13.4)	30 (16.3)	14 (10.9)
Ran into stationary object	16 (5.5)	12 (6.5)	7 (5.4)
Sport	156 (53.6)	93 (50.5)	67 (51.9)
Other mechanisms of injury	58 (19.9)	35 (19.0)	29 (22.5)
Loss of consciousness	48 (16.5)	32 (17.4)	20 (15.5)
<1 min	38 (79.2)	24 (75.0)	14 (70.0)
1-5 min	8 (16.7)	7 (21.9)	5 (25.0)
>5 min	2 (4.2)	1 (3.1)	1 (5.0)

Abbreviations: 5P, Predicting Persistent Postconcussive Problems in Pediatrics.

^a^ Two participants did not have values of time from injury to triage.

**Table 2.** Participant Sleep Habits Throughout 2 Weeks of Acute Recovery

Variable	Median (IQR)	
	Nights 1-7	Nights 1-14
Sleep duration, h	10.0 (9.0-11.0)	9.9 (8.9-10.8)
Weekday night	10.0 (9.0-11.0)	9.8 (8.8-10.5)
Weekend night	10.5 (9.3-11.3)	10.0 (9.1-11.0)
Bedtime	22:00 (21:00-23:00)	22:00 (21:10-23:00)
Weekday night	22:00 (21:00-22:45)	22:00 (21:00-23:00)
Weekend night	22:04 (21:30-23:00)	22:30 (21:30-23:20)
Waketime	8:00 (7:00-9:00)	7:45 (7:00-9:00)
Weekday morning	7:30 (6:52-8:51)	7:30 (6:45-8:30)
Weekend morning	8:30 (7:40-9:30)	8:30 (7:30-9:30)
Sleep midpoint	3:00 (2:15-3:45)	2:58 (2:15-3:45)
Weekday night	2:45 (2:08-3:36)	2:45 (2:05-3:30)
Weekend night	3:20 (2:45-4:15)	3:30 (2:45-4:15)

**Figure 1.**
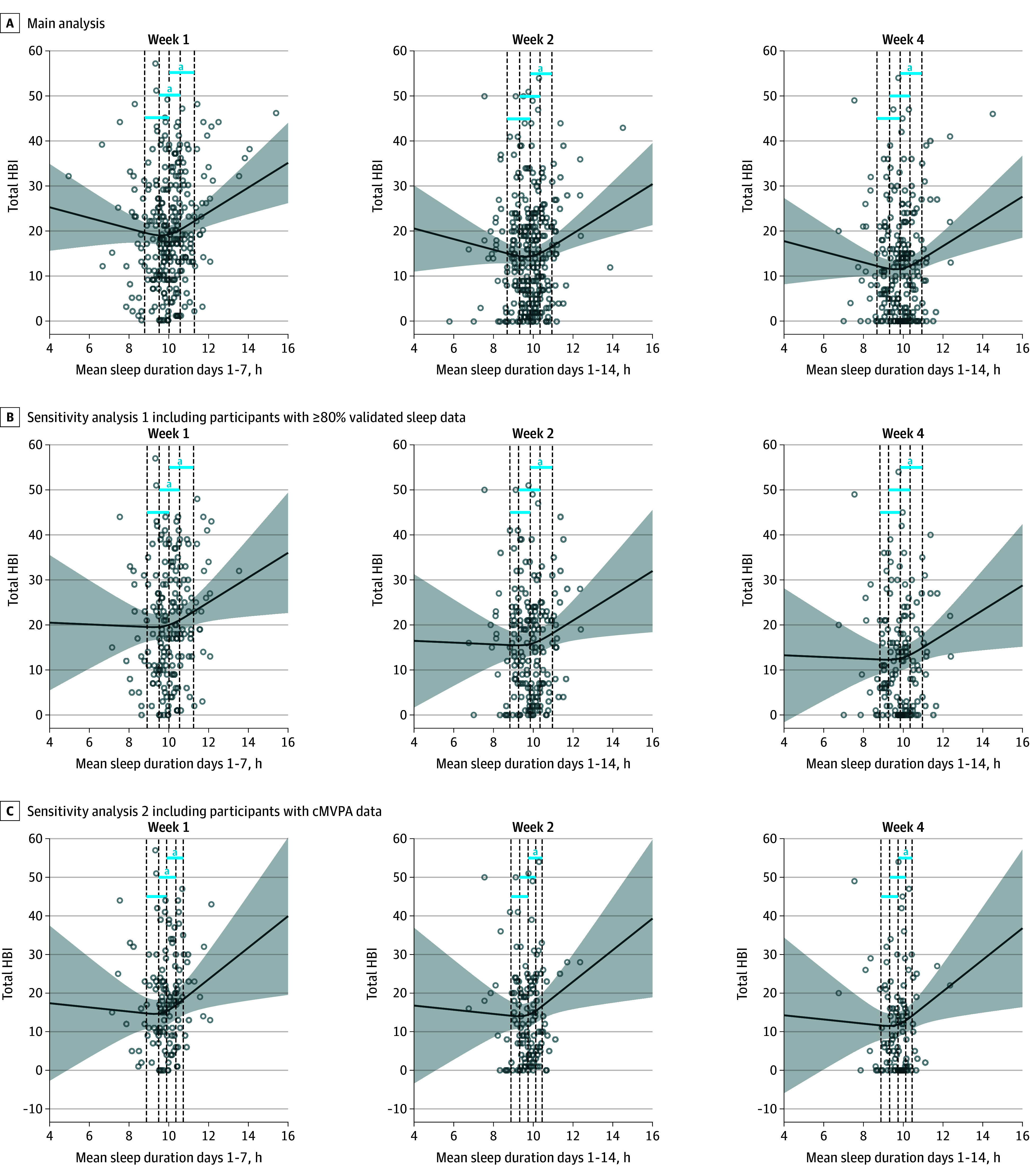
Association Between Nightly Mean Sleep Duration and Symptom Burden at 1, 2, and 4 Weeks Postconcussion A, Included 291 participants; B, included 184 participants; C, included 129 participants. Dashed vertical lines indicate (left to right) 10th, 25th, 50th, 75th, and 90th percentiles of mean sleep duration. Curves are adjusted for age, sex, baseline Health and Behavior Inventory (HBI), retrospective HBI, randomization group, Predicting Persistent Postconcussive Problems in Pediatrics score, diagnostic history, pre-injury tired rating, and pre-injury drowsiness rating. Points on the graph represent observed data (HBI vs mean sleep duration). Shaded areas represent the 95% CIs. ^a^Contrast significance (*P* < .05).

The 10th vs 50th percentile difference of mean sleep duration was nonsignificant at 1 week (8.8 hours vs 10.0 hours: estimate, −0.40 [95% CI, −1.97 to 1.17]; *P* = .62), 2 weeks (8.7 hours vs 9.9 hours: estimate, −0.66 [95% CI, −2.30 to 0.97]; *P* = .43), and 4 weeks (estimate, −0.66 [95% CI, −2.30 to 0.97]; *P* = .43) (Figure 1A). The 25th vs 75th percentile difference of mean sleep duration was significant at 1 week (9.5 hours vs 10.6 hours: estimate, 1.26 [95% CI, 0.25 to 2.28]; *P* = .02), but not at 2 weeks (9.3 hours vs 10.3 hours: estimate, 0.66 [95% CI, −0.33 to 1.64]; *P* = .19) or 4 weeks (estimate, 0.66 [95% CI, −0.33 to 1.64]; *P* = .19) (Figure 1A). The 50th vs 90th percentile difference of mean sleep duration was significant at 1 week (10.0 hours vs 11.3 hours: estimate, 2.96 [95% CI, 1.22 to 4.69]; *P* < .001), 2 weeks (9.9 hours vs 10.9 hours: estimate, 2.16 [95% CI, 0.85 to 3.47]; *P* = .001), and 4 weeks (estimate, 2.16 [95% CI, 0.85 to 3.47]; *P* = .001) (Figure 1A).

The first sensitivity analysis included 184 of 291 participants who had both overnight accelerometer data and a sleep log for 80% or more of their complete days of sleep data (Table 1). The sensitivity analysis yielded similar contrast results to the main analysis (Figure 1B; eTable 2 in Supplement 1). The second sensitivity analysis included 129 of 291 participants who had valid cumulative MVPA data for at least the first week (Table 1). The sensitivity analysis yielded similar contrast results to the main analysis (Figure 1C; eTable 3 in Supplement 1).

A total of 286 participants were included in the logistic regression at 2 weeks postconcussion. Of these, 37 participants (12.9%) were reliably symptomatic using the conservative definition (*z* ≥ 1.65) and 55 participants (19.2%) were reliably symptomatic using the liberal definition (*z* ≥ 1.28) (eFigure 3 in Supplement 1). Mean sleep duration was nonsignificant in the 2-week models, as were all percentile difference contrasts (Table 3).

**Table 3.** Odds Ratios for Each of the Logistic Regressions Contrasts at 2 and 4 Weeks

*z* Score threshold	Odds ratio (95% CI)	*P* value	Whole model *P* value
2 wk (n = 286)			
1.65			
10th vs 50th percentile	0.92 (0.46-1.84)	.82	<.001
25th vs 75th percentile	1.10 (0.75-1.60)	.63	
50th vs 90th percentile	1.32 (0.80-2.18)	.28	
1.28			
10th vs 50th percentile	1.46 (0.72-2.92)	.29	<.001
25th vs 75th percentile	1.35 (0.94-1.95)	.11	
50th vs 90th percentile	1.33 (0.84-2.10)	.23	
**4 wk (n = 221)**			
1.65			
10th vs 50th percentile	0.98 (0.43-2.26)	.96	<.001
25th vs 75th percentile	1.24 (0.75-2.04)	.41	
50th vs 90th percentile	1.73 (0.91-3.26)	.09	
1.28			
10th vs 50th percentile	0.90 (0.44-1.81)	.76	<.001
25th vs 75th percentile	1.23 (0.80-1.88)	.34	
50th vs 90th percentile	1.93 (1.07-3.47)	.03	

A total of 221 participants were included in the logistic regression at 4 weeks postconcussion. Of these, 24 participants (10.9%) were reliably symptomatic using the conservative definition and 34 participants (15.4%) were reliably symptomatic using the liberal definition (eFigure 4 in Supplement 1). Mean sleep duration over 2 weeks was nonsignificant in the 4-week models. At 4 weeks, the 50th (9.9 hours) vs 90th (10.9 hours) percentile difference contrast was nonsignificant in the conservative model (odds ratio [OR], 1.73 [95% CI, 0.91-3.26]; *P* = .09), but was significant in the liberal model (OR, 1.93 [95% CI, 1.07-3.47]; *P* = .03) (Table 3). The remaining contrasts were nonsignificant at 4 weeks. When compared with null models, the adjusted logistic regression models were statistically significant (*P* < .001).

**Figure 2.**
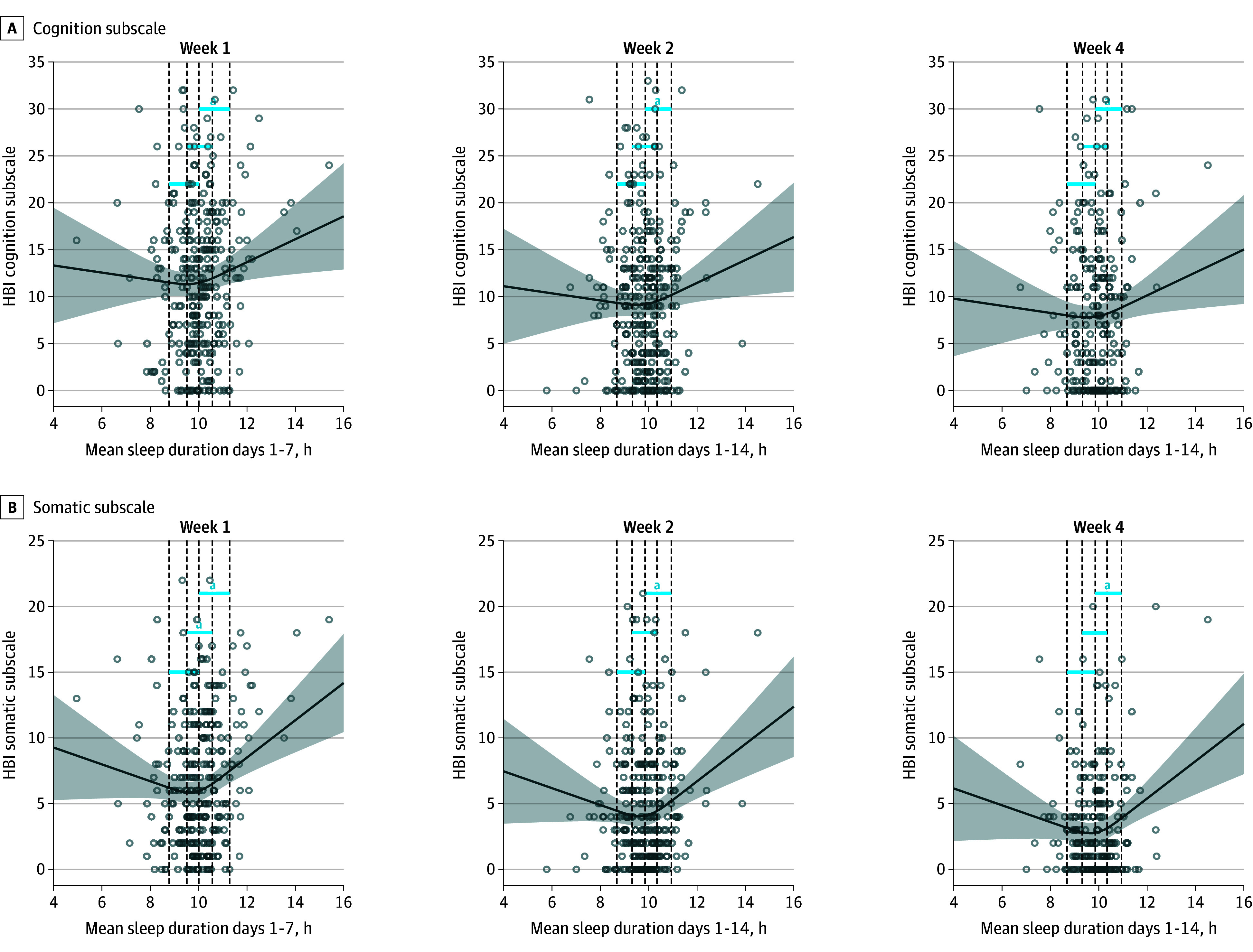
Association Between Mean Sleep Duration and Cognitive and Somatic Symptom Burden at 1, 2, and 4 Weeks Postconcussion A, This exploratory analysis included 290 participants; B, this exploratory analysis included 290 participants. Dashed lines indicate (left to right) 10th, 25th, 50th, 75th, and 90th percentiles. Curves are adjusted for age, sex, baseline cognitive or somatic Health and Behavior Inventory (HBI), retrospective cognitive of somatic HBI, randomization group, Predicting Persistent Postconcussive Problems in Pediatrics score, diagnostic history, pre-injury tired rating, and pre-injury drowsiness rating. Shaded areas represent the 95% CIs. ^a^Contrast significance (*P* < .05).

A total of 290 of 456 participants were included in each of the exploratory analyses evaluating cognitive and somatic symptoms separately. Contrast results for the exploratory analyses were similar to the main analysis; however, the 25th (9.5 hours) vs 75th (10.6 hours) percentile difference contrast for the cognitive symptom subscale was nonsignificant at 1 week (eTable 4 in Supplement 1; Figure 2).

## Discussion

Among youths with acute concussion, more sleep during the first week (ie, over 9.5 hours per day) or during the first 2 weeks (ie, over 9.9 hours per day) was associated with higher symptom burden. The model curves presented a possible U-shaped association between sleep and symptoms, although the contrasts at the lower end (10th vs 50th percentile difference) were nonsignificant. Within this sample, mean sleep duration beyond the 50th percentile during the first 2 weeks after concussion was associated with higher total, cognitive, and somatic symptom burden at 1, 2, and 4 weeks postinjury.

According to the Canadian 24-Hour Movement Guidelines for Children and Youth^9^ and American National Sleep Foundation,^50^ youths aged 10 to 13 years and 14 to 17 years should have a total uninterrupted sleep duration of 9 to 11 hours and 8 to 10 hours per night, respectively. In general, our sample met the recommended sleep duration, with a 10th percentile of 8.7 hours and a 90th percentile of 10.9 hours over the first 2 weeks of recovery. The median sleep duration of 10 hours falls in the middle-to-long range based on the Canadian^9^ and American^50^ sleep guidelines for this age group, and are considered long when compared to an American epidemiological study where youths aged 6 to 13 years and 13 to 17 years had median sleep durations of 8.97 hours and 8.29 hours, respectively.^51^ Given that increased sleep has been identified as a common outcome following pediatric concussion,^17^ the observed long sleep durations are not unexpected, although they were still within the healthy range. Sleep duration estimates were relatively similar between days 1 to 7 and days 1 to 14. Median bedtimes and waketimes were later on weekends compared with weekdays.

Our findings of mean sleep duration beyond 9.9 hours associated with higher symptom burden contrasts other findings in pediatric concussion research where short sleep durations have been associated with higher symptom burden and prolonged recovery,^18,21,22,23^ or the associations were nonsignificant.^24,25,26,27,28,29^ Pediatric concussion studies with wrist-worn activity monitors have reported varying mean or median sleep durations ranging from 6.9 to 8.6 hours in their samples.^23,24,25,26,28,29,30,31,52^ These sleep durations are shorter than our 10th percentiles of 8.7 to 8.9 hours, which may explain the inconsistent results. However, Kostyun et al^22^ observed that sleeping more than usual and longer than 9 hours before neurocognitive testing was associated with reduced scores beyond the acute stage of sport-related concussion in youths, which is partially consistent with our results.

In addition to sleep, daily behaviors such as MVPA,^41^ attending school,^53^ and screen time^54^ have each been previously investigated in relation to symptom burden. Interestingly, increased MVPA over the first 2 weeks of recovery predicted reduced symptom burden at 2 weeks postconcussion.^41^ In addition, early return to school following pediatric concussion has been associated with reduced symptom burden at 2 weeks,^53^ and better sleep quality throughout recovery.^14^ If youths with concussion are spending less time in bed because they are attending school and involved in other activities and behaviors,^7,9^ then we may hypothesize that the observed association between sleep and symptoms is affected by these activities during the day. However, when we included cumulative MVPA in our model, the association of long sleep durations (over 9.9 hours) with increased symptoms remained significant. This suggests that youths with concussion who spend long hours in bed may experience higher symptom burden regardless of how much time they spend engaging in MVPA.

A previous study reported that recovery times for concussed participants with sleep disturbances were 3 to 4 times longer than those for participants without sleep disturbances.^14,15^ A bidirectional relationship has been suggested, where sleep disturbances are a result of concussion and also prolong recovery.^16,31,55,56^ The significance values in our exploratory analyses indicated that mean sleep duration was associated with a greater likelihood of somatic symptoms (such as headache, dizziness, tiredness, feeling faint^43^) than with cognitive symptoms, suggesting that those with higher somatic symptoms may be spending more time in bed. The HBI includes 2 items in the somatic subscale related to fatigue, “I get tired easily” and “I get tired a lot,”^43,44^ which may explain the difference. Alternatively, participants may be spending more time in bed due to fear avoidance, which is thought to be associated with acute^57^ and chronic postconcussive symptoms in adults.^58^ Further research should prioritize investigating whether increased sleep duration leads to increased symptoms, or whether the increased symptoms lead to increased time in bed and reduced time spent participating in other daily activities that could benefit recovery (eg, school,^53^ MVPA^41^).

Finally, the mean sleep duration variable was not associated with reliable change in symptoms at 2 or 4 weeks. These results are consistent with previous studies, where objectively measured sleep duration was not associated with persistent symptoms at 4 to 6 weeks postinjury.^24,26,28^ However, we observed participants who slept 10.9 hours over 2 weeks had significantly greater odds of remaining symptomatic at 4 weeks, compared with those who slept 9.9 hours, in the liberal model but not the conservative model. This suggests that those who sleep greater than 9.9 hours over the first 2 weeks of concussion may have increased odds of PSAC. These statistical discrepancies may have been influenced by the small group size of reliably symptomatic youths at 2 and 4 weeks. Additional analyses with larger samples of reliably symptomatic youths are required to verify these findings.

### Strengths and Limitations

Strengths of the current study include the large sample (more than 220 participants), the inclusion of diverse mechanisms of injury, the use of objective movement data, and longitudinal design. Previous systematic reviews on sleep and concussion have heavily relied on self- and parent-reported subjective sleep measures, with evidence far outweighing more objective device-based sleep measures.^17,59,60,61^ The reliance on self- or proxy-reported measures for knowledge synthesis introduces a risk of bias, making the inclusion of the accelerometer data to confirm self-reported bedtimes and waketimes a significant strength of the current study. In our sensitivity analysis, we included participants with at least 80% of confirmed data. This conservative threshold retained 63% of participants and confirmed the findings from the main analysis. Furthermore, the 2-week longitudinal study design allowed investigations into the patterns and implications of daily sleep during the first week of recovery and the combined first 2 weeks of recovery. This acute timeline is essential for investigating prognostic elements of brain injury.

This study also had several limitations. Our sample had missing data. Of the original 456 PedCARE participants, 61 did not have the minimum required sleep data and another 104 did not have the minimum required outcome variables or covariates for inclusion in the main mixed-effects model. Given the daily commitment required for 2-week longitudinal sleep measures, our sample may be biased to include the most motivated participants.

This study assessed symptom burden and reliable change in symptoms with the HBI. Although the HBI is an National Institutes of Health Common Data Element for Concussion^62^ and Pediatric Traumatic Brain Injury,^63^ it is not validated in youths older than 16.99 years of age.^44^ The current study includes participants aged 10 to 17.99 years of age, although only 16 participants (5.5%) were older than 17 years.

Participants were recruited from 3 Canadian pediatric EDs, which may introduce sampling bias. Youths with concussion may seek care outside of the ED or not at all, leading to a sample that is not generalized.

Our study included a waist-worn accelerometer, which is both a strength and a limitation. The waist-worn accelerometer does not explicitly identify periods of sleep onset or offset, but instead records activity counts per minute, which can be used to estimate or assist with the estimation of sleep values. These activity counts were used to confirm or validate bedtimes and waketimes recorded in daily sleep logs and estimate these values when logs were incomplete. Due to the nature of the waist-worn accelerometer, we were unable to objectively assess sleep efficiency, therefore it is possible that the participants were not asleep for the entire duration from bedtime to waketime.

Given that the primary focus of the PedCARE study was physical activity, data on screentime, nap time, history of personal vs familial sleep disorders, and use of medication or melatonin were not collected. Naps and daytime sleep are common in the youth population and have been evaluated in other concussion studies.^31,64^ Future studies should replicate our well-adjusted analyses with a larger sample, daytime sleep, diverse mechanisms of injury, prognostically important covariates, and actigraphy over the first 2-plus weeks of pediatric concussion recovery.

## Conclusions

The Living Guidelines for Pediatric Concussion Care recommends that youths engage in relative rest for 24 to 48 hours postinjury and maintain a regular, sufficient sleep schedule during recovery.^65,66^ In this observational study of youths with acute concussion, long sleep durations during the first 2 weeks postconcussion (ie, over 9.9 hours) were associated with more symptoms at 1, 2, and 4 weeks, even when controlling for cumulative MVPA during the first 2 weeks among other prognostically important variables. Furthermore, long sleep duration may be associated with increased odds of being reliably symptomatic at 4 weeks, therefore a greater risk of PSAC. Clinicians should monitor youths’ sleep after concussion.

## Notes

Supplement 1.
eMethods.
**eFigure 1.** Participation Flowchart for the Main Analysis and Sensitivity Analyses **eTable 1.** Percentiles of Mean Sleep Duration Over the First Week and First Two Weeks of Recovery **eFigure 2.** LOESS Curves of Observed Health and Behaviour Inventory (HBI) vs Mean Sleep Duration at 1-, 2-, and 4-Weeks Indicate Nonlinearity **eTable 2.** Contrast Results for the Sensitivity Analysis 1 With Confirmed Sleep Data **eTable 3.** Contrast Results for the Sensitivity Analysis 2 With Additional Time-Varying cMVPA Variable **eFigure 3.** Distribution of Mean Sleep Durations for Reliably Symptomatic and Asymptomatic Participants at 2-Weeks Postconcussion **eFigure 4.** Distribution of Mean Sleep Durations for Reliably Symptomatic and Asymptomatic Participants at 4-Weeks Postconcussion **eTable 4.** Contrast Results for the Exploratory Analyses With the Cognition and Somatic Symptom Subscales

Supplement 2.
Nonauthor Collaborators

Supplement 3.
Data Sharing Statement

## References

[1] Chadwick L, Sharma MJ, Madigan S, Callahan BL, Owen Yeates K. Classification criteria and rates of persistent postconcussive symptoms in children: a systematic review and meta-analysis. J Pediatr. 2022;246:131-137.e2. doi:10.1016/j.jpeds.2022.03.039 35358589

[2] Zemek R, Barrowman N, Freedman SB, ; Pediatric Emergency Research Canada (PERC) Concussion Team. Clinical risk score for persistent postconcussion symptoms among children with acute concussion in the ED. JAMA. 2016;315(10):1014-1025. doi:10.1001/jama.2016.1203 26954410

[3] Broshek DK, Pardini JE, Herring SA. Persisting symptoms after concussion: time for a paradigm shift. PM R. 2022;14(12):1509-1513. doi:10.1002/pmrj.12884 36152344 PMC10087676

[4] Novak Z, Aglipay M, Barrowman N, ; Pediatric Emergency Research Canada Predicting Persistent Postconcussive Problems in Pediatrics (PERC 5P) Concussion Team. Association of persistent postconcussion symptoms with pediatric quality of life. JAMA Pediatr. 2016;170(12):e162900. doi:10.1001/jamapediatrics.2016.2900 27775762

[5] Ledoux AA, Webster RJ, Clarke AE, . Risk of mental health problems in children and youths following concussion. JAMA Netw Open. 2022;5(3):e221235. doi:10.1001/jamanetworkopen.2022.1235 35254429 PMC8902648

[6] Bang F, Roberts KC, Chaput JP, Goldfield GS, Prince SA. Physical activity, screen time and sleep duration: combined associations with psychosocial health among Canadian children and youth. Health Rep. 2020;31(5):9-16. doi:10.25318/82-003-X202000500002-ENG 32644766

[7] Chaput JP, Gray CE, Poitras VJ, . Systematic review of the relationships between sleep duration and health indicators in school-aged children and youth. Appl Physiol Nutr Metab. 2016;41(6)(suppl 3):S266-S282. doi:10.1139/apnm-2015-0627 27306433

[8] Dutil C, Podinic I, Sadler CM, . Sleep timing and health indicators in children and adolescents: a systematic review. Health Promot Chronic Dis Prev Can. 2022;42(4):150-169. doi:10.24095/hpcdp.42.4.04 35481337 PMC9116724

[9] Tremblay MS, Carson V, Chaput JP, . Canadian 24-hour movement guidelines for children and youth: an integration of physical activity, sedentary behaviour, and sleep. Appl Physiol Nutr Metab. 2016;41(6)(suppl 3):S311-S327. doi:10.1139/apnm-2016-0151 27306430

[10] Ojio Y, Nishida A, Shimodera S, Togo F, Sasaki T. Sleep duration associated with the lowest risk of depression/anxiety in adolescents. Sleep. 2016;39(8):1555-1562. doi:10.5665/sleep.6020 27306271 PMC4945315

[11] Winsler A, Deutsch A, Vorona RD, Payne PA, Szklo-Coxe M. Sleepless in Fairfax: the difference one more hour of sleep can make for teen hopelessness, suicidal ideation, and substance use. J Youth Adolesc. 2015;44(2):362-378. doi:10.1007/s10964-014-0170-3 25178930

[12] Wolfson AR, Carskadon MA. Sleep schedules and daytime functioning in adolescents. Child Dev. 1998;69(4):875-887. doi:10.1111/j.1467-8624.1998.tb06149.x 9768476

[13] Beebe DW, Powers SW, Slattery EW, Gubanich PJ. Short sleep and adolescents’ performance on a concussion assessment battery: an experimental sleep manipulation study. Clin J Sport Med. 2018;28(4):395-397. doi:10.1097/JSM.0000000000000454 28742612 PMC5776054

[14] Fisher M, Wiseman-Hakes C, Obeid J, DeMatteo C. Does sleep quality influence recovery outcomes after postconcussive injury in children and adolescents? J Head Trauma Rehabil. 2023;38(3):240-248. doi:10.1097/HTR.0000000000000811 35997760

[15] Bramley H, Henson A, Lewis MM, Kong L, Stetter C, Silvis M. Sleep disturbance following concussion is a risk factor for a prolonged recovery. Clin Pediatr (Phila). 2017;56(14):1280-1285. doi:10.1177/0009922816681603 29073787

[16] Luszawski CA, Minich NM, Bigler ED, . Sleep disturbance and postconcussive symptoms in pediatric mild traumatic brain injury and orthopedic injury. J Head Trauma Rehabil. Published online September 13, 2024. doi:10.1097/HTR.0000000000001005 39808542

[17] Djukic S, Phillips NL, Lah S. Sleep outcomes in pediatric mild traumatic brain injury: a systematic review and meta-analysis of prevalence and contributing factors. Brain Inj. 2022;36(12-14):1289-1322. doi:10.1080/02699052.2022.2140198 36413091

[18] Cassimatis M, Orr R, Fyffe A, Browne G. Association of sleep disturbance with neurocognition, symptom severity, and recovery in pediatric concussion: a 10-year retrospective analysis of a tertiary referral concussion clinic. J Head Trauma Rehabil. 2023;38(3):231-239. doi:10.1097/HTR.0000000000000804 35862900

[19] Chung JS, Zynda AJ, Didehbani N, . Association between sleep quality and recovery following sport-related concussion in pediatrics. J Child Neurol. 2019;34(11):639-645. doi:10.1177/0883073819849741 31113274

[20] Tham SW, Fales J, Palermo TM. Subjective and objective assessment of sleep in adolescents with mild traumatic brain injury. J Neurotrauma. 2015;32(11):847-852. doi:10.1089/neu.2014.3559 25707446 PMC4449620

[21] Hrabarchuk EI, Kalagara R, Ezzat B, . Effects of hours of sleep on ImPACT concussion testing: comparing baseline with postinjury scores. J Neurosurgery. 2024;34(2):121-128. doi:10.3171/2024.2.PEDS23437 38701519

[22] Kostyun RO, Milewski MD, Hafeez I. Sleep disturbance and neurocognitive function during the recovery from a sport-related concussion in adolescents. Am J Sports Med. 2015;43(3):633-640. doi:10.1177/0363546514560727 25520301

[23] Trbovich AM, Howie EK, Elbin RJ, . The relationship between accelerometer-measured sleep and next day ecological momentary assessment symptom report during sport-related concussion recovery. Sleep Health. 2021;7(4):519-525. doi:10.1016/j.sleh.2021.03.006 33933377

[24] Barlow KM, Girgulis KA, Goldstein G, . Sleep parameters and overnight urinary melatonin production in children with persistent post-concussion symptoms. Pediatr Neurol. 2020;105:27-34. doi:10.1016/j.pediatrneurol.2019.11.006 32029332

[25] Brayton RP, Price AM, Jones C, Ellis C, Burkhart S, Knell G. Prospective evaluation of 24-hour movement behaviors among adolescents recovering from a sport-related concussion. Applied Neuropsychology: Child. 2024;24(4):334-342. doi:10.1080/21622965.2023.2181082 36809228

[26] Fisher M, Wiseman-Hakes C, Obeid J, DeMatteo C. Examining the trajectory and predictors of post-concussion sleep quality in children and adolescents. Brain Inj. 2022;36(2):166-174. doi:10.1080/02699052.2022.2043439 35213283

[27] Murdaugh DL, Ono KE, Reisner A, Burns TG. Assessment of sleep quantity and sleep disturbances during recovery from sports-related concussion in youth athletes. Arch Phys Med Rehabil. 2018;99(5):960-966. doi:10.1016/j.apmr.2018.01.005 29425698

[28] Neely LM, Smulligan KL, Wingerson MJ, . The association between sleep and physical activity with persisting postconcussion symptoms among adolescent athletes. PM R. 2023;15(9):1122-1129. doi:10.1002/pmrj.12939 36580488 PMC10875599

[29] Tham SW, Aaron RV, Palermo TM. The role of sleep deficiency in the trajectory of postconcussive symptoms in adolescents. Brain Inj. 2019;33(11):1413-1419. doi:10.1080/02699052.2019.1643921 31322003 PMC7243849

[30] Sufrinko AM, Howie EK, Elbin RJ, Collins MW, Kontos AP. A preliminary investigation of accelerometer-derived sleep and physical activity following sport-related concussion. J Head Trauma Rehabil. 2018;33(5):E64-E74. doi:10.1097/HTR.0000000000000387 29601343

[31] VonDeylen O, Alshaikh E, Wheeler K, . Sleep quantity and quality during the first week postinjury and time to symptom resolution in youth with concussion. Br J Sports Med. 2024;59(10):109058. doi:10.1136/bjsports-2024-109058 PMC13355237 40011013

[32] Borghese MM, Lin Y, Chaput JP, Janssen I. Estimating sleep efficiency in 10- to- 13-year-olds using a waist-worn accelerometer. Sleep Health. 2018;4(1):110-115. doi:10.1016/j.sleh.2017.09.006 29332671

[33] Borghese MM. *Advances in Objectively Measured Movement Behaviours in Children*. Master’s thesis. Queen’s University; 2018. Accessed September 19, 2023. https://qspace.library.queensu.ca/items/eb3e8ae4-6719-414d-991b-2b7cb852e448

[34] Callender LK, Borghese MM, Janssen I. Which intensities, types, and patterns of movement behaviors are most strongly associated with cardiometabolic risk factors among children? J Sport Health Sci. 2021;10(3):368-378. doi:10.1016/j.jshs.2019.06.006 33993923 PMC8167329

[35] Borghese MM, Tremblay MS, LeBlanc AG, Leduc G, Boyer C, Chaput JP. Comparison of ActiGraph GT3X+ and Actical accelerometer data in 9–11-year-old Canadian children. J Sports Sci. 2017;35(6):517-524. doi:10.1080/02640414.2016.1175653 27103499

[36] Ledoux AA, Barrowman NJ, Boutis K, ; Pediatric Emergency Research Canada PedCARE team. Multicentre, randomised clinical trial of paediatric concussion assessment of rest and exertion (PedCARE): a study to determine when to resume physical activities following concussion in children. Br J Sports Med. 2019;53(3):195-195. doi:10.1136/bjsports-2017-097981 28701360

[37] Ledoux AA, Barrowman N, Bijelić V, ; PERC PedCARE Concussion team. Is early activity resumption after paediatric concussion safe and does it reduce symptom burden at 2 weeks post injury? The Pediatric Concussion Assessment of Rest and Exertion (PedCARE) multicentre randomised clinical trial. Br J Sports Med. 2022;56(5):271-278. doi:10.1136/bjsports-2021-105030 34836880

[38] McCrory P, Meeuwisse WH, Aubry M, . Consensus statement on concussion in sport: the 4th International Conference on Concussion in Sport held in Zurich, November 2012. Br J Sports Med. 2013;47(5):250-258. doi:10.1136/bjsports-2013-092313 23479479

[39] McCrory P, Meeuwisse W, Dvorak J, . Consensus statement on concussion in sport—the 5th international conference on concussion in sport held in Berlin, October 2016. Brit J Sports Med. 2017;51(11):097699. doi:10.1136/bjsports-2017-097699 28446457

[40] Peterson A, Gabella BA, Johnson J, . Multisite medical record review of emergency department visits for unspecified injury of head following the ICD-10-CM coding transition. Inj Prev. 2021;27(S1):i13-i18. doi:10.1136/injuryprev-2019-043517 33674328 PMC7948189

[41] Ledoux AA, Sicard V, Bijelić V, ; PERC PedCARE team. Optimal volume of moderate-to-vigorous physical activity postconcussion in children and adolescents. JAMA Netw Open. 2024;7(2):e2356458. doi:10.1001/jamanetworkopen.2023.56458 38363567 PMC10873766

[42] Guskiewicz KM. Assessment of postural stability following sport-related concussion. Curr Sports Med Rep. 2003;2(1):24-30. doi:10.1249/00149619-200302000-00006 12831673

[43] Ayr LK, Yeates KO, Taylor HG, Browne M. Dimensions of postconcussive symptoms in children with mild traumatic brain injuries. J Int Neuropsychol Soc. 2009;15(1):19-30. doi:10.1017/S1355617708090188 19128525 PMC2832119

[44] Zhang C, Tang K, Zemek R, . Factor structure and measurement invariance of post-concussion symptom ratings on the Health and Behaviour Inventory across time, raters, and groups: an A-CAP study. J Int Neuropsychol Soc. 2022;29(4):346-359. doi:10.1017/S1355617722000340 35924559

[45] O’Brien H, Minich NM, Langevin LM, . Normative and psychometric characteristics of the health and behavior inventory among children with mild orthopedic injury presenting to the emergency department: Implications for assessing postconcussive symptoms using the Child Sport Concussion Assessment Tool 5th Edition (Child SCAT5). Clin J Sport Med. 2021;31(5):e221-e228. doi:10.1097/JSM.0000000000000943 33973883 PMC8416708

[46] Harris PA, Taylor R, Thielke R, Payne J, Gonzalez N, Conde JG. Research electronic data capture (REDCap)–a metadata-driven methodology and workflow process for providing translational research informatics support. J Biomed Inform. 2009;42(2):377-381. doi:10.1016/j.jbi.2008.08.010 18929686 PMC2700030

[47] Harris PA, Taylor R, Minor BL, ; REDCap Consortium. The REDCap consortium: building an international community of software platform partners. J Biomed Inform. 2019;95:103208. doi:10.1016/j.jbi.2019.103208 31078660 PMC7254481

[48] Sady MD, Vaughan CG, Gioia GA. Psychometric characteristics of the postconcussion symptom inventory in children and adolescents. Arch Clin Neuropsychol. 2014;29(4):348-363. doi:10.1093/arclin/acu014 24739735 PMC4030704

[49] Teel EF, Zemek RL, Tang K, ; Pediatric Emergency Research Canada (PERC) Concussion Team. The stability of retrospective pre-injury symptom ratings following pediatric concussion. Front Neurol. 2019;10:672. doi:10.3389/fneur.2019.00672 31316452 PMC6610489

[50] Hirshkowitz M, Whiton K, Albert SM, . National Sleep Foundation’s sleep time duration recommendations: methodology and results summary. Sleep Health. 2015;1(1):40-43. doi:10.1016/j.sleh.2014.12.010 29073412

[51] Su S, Li X, Xu Y, McCall WV, Wang X. Epidemiology of accelerometer-based sleep parameters in US school-aged children and adults: NHANES 2011-2014. Sci Rep. 2022;12(1):7680. doi:10.1038/s41598-022-11848-8 35538108 PMC9090869

[52] Maerlender A, Masterson C, Calvi JL, Caze T, Mathiasen R, Molfese D. Sleep and stress in the acute phase of concussion in youth. Sports Med Health Sci. 2020;2(2):109-114. doi:10.1016/j.smhs.2020.06.003 35784179 PMC9219335

[53] Vaughan CG, Ledoux AA, Sady MD, ; PERC 5P Concussion Team. Association between early return to school following acute concussion and symptom burden at 2 weeks postinjury. JAMA Netw Open. 2023;6(1):e2251839. doi:10.1001/jamanetworkopen.2022.51839 36662524 PMC9860528

[54] Cairncross M, Yeates KO, Tang K, . Early postinjury screen time and concussion recovery. Pediatrics. 2022;150(5):e2022056835. doi:10.1542/peds.2022-056835 36250231

[55] Hughes C, Hunt K, Cox B, Raybon J, Lopez RM. Sleep dysfunction in adolescents with prolonged postconcussion symptoms: a reciprocal coupling of traumatic brain injury and sleep-related problems. J Sport Rehabil. 2022;31(6):809-814. doi:10.1123/jsr.2021-0277 35365589

[56] Wickwire EM, Williams SG, Roth T, . Sleep, sleep disorders, and mild traumatic brain injury. what we know and what we need to know: findings from a national working group. Neurotherapeutics. 2016;13(2):403-417. doi:10.1007/s13311-016-0429-3 27002812 PMC4824019

[57] Patlan I, Gamelin G, Khalaj K, Castonguay T, Dover G. Athlete fear avoidance, depression, and anxiety are associated with acute concussion symptoms in athletes. J Clin Med. 2024;13(8):2401. doi:10.3390/jcm13082401 38673675 PMC11050826

[58] Wijenberg MLM, Stapert SZ, Verbunt JA, Ponsford JL, Van Heugten CM. Does the fear avoidance model explain persistent symptoms after traumatic brain injury? Brain Inj. 2017;31(12):1597-1604. doi:10.1080/02699052.2017.1366551 28980825

[59] Botchway EN, Godfrey C, Anderson V, Catroppa C. A Systematic review of sleep-wake disturbances in childhood traumatic brain injury: relationship with fatigue, depression, and quality of life. J Head Trauma Rehabil. 2019;34(4):241-256. doi:10.1097/HTR.0000000000000446 30499928

[60] Ludwig R, D’Silva L, Vaduvathiriyan P, Rippee MA, Siengsukon C. Sleep disturbances in the acute stage of concussion are associated with poorer long-term recovery: a systematic review. PM R. 2020;12(5):500-511. doi:10.1002/pmrj.12309 31876086

[61] Ludwig R, Nelson E, Vaduvathiriyan P, Rippee MA, Siengsukon C. Sleep quality in the chronic stage of concussion is associated with poorer recovery: a systematic review. J Concussion. 2021;5. doi:10.1177/20597002211020881 31876086

[62] Broglio SP, Kontos AP, Levin H, . National Institute of Neurological Disorders and Stroke and Department of Defense Sport-Related Concussion common data elements version 1.0 recommendations. J Neurotrauma. 2018;35(23):2776-2783. doi:10.1089/neu.2018.5643 29717643 PMC6247979

[63] McCauley SR, Wilde EA, Anderson VA, ; Pediatric TBI Outcomes Workgroup. Recommendations for the use of common outcome measures in pediatric traumatic brain injury research. J Neurotrauma. 2012;29(4):678-705. doi:10.1089/neu.2011.1838 21644810 PMC3289848

[64] Wiseman-Hakes C, Gosselin N, Sharma B, Langer L, Gagnon I. A longitudinal investigation of sleep and daytime wakefulness in children and youth with concussion. ASN Neuro. Published online January 10, 2019. doi:10.1177/1759091418822405 30806074 PMC6343438

[65] Dawson J, Reed N, Bauman S, Seguin R, Zemek R. Diagnosing and managing paediatric concussion: Key recommendations for general paediatricians and family doctors. Paediatr Child Health. 2021;26(7):402-407. doi:10.1093/pch/pxab024 34777657 PMC8581524

[66] Reed N, Zemek R, Dawson J, . Living guideline for pediatric concussion care. Accessed December 3, 2024. http://www.pedsconcussion.com

