# Supplementary material for: Nightly Sleep Duration and Symptom Burden Over 1 Month Following Pediatric Concussion
Source: JAMA Netw Open. 2025 Jun 18;8(6):e2516333. doi: 10.1001/jamanetworkopen.2025.16333 (PMC12177668; doi:10.1001/jamanetworkopen.2025.16333)
Supplement: Supplement 1. — eMethods. eFigure 1. Participation Flowchart for the Main Analysis and Sensitivity Analyses eTable 1. Percentiles of Mean Sleep Duration Over the First Week and First Two Weeks of Recovery eFigure 2. LOESS Curves of Observed Health and Behaviour Inventory (HBI) vs Mean Sleep Duration at 1-, 2-, and 4-Weeks Indicate Nonlinearity eTable 2. Contrast Results for the Sensitivity Analysis 1 With Confirmed Sleep Data eTable 3. Contrast Results for the Sensitivity Analysis 2 With Additional Time-Varying cMVPA Variable eFigure 3. Distribution of Mean Sleep Durations for Reliably Symptomatic and Asymptomatic Participants at 2-Weeks Postconcussion eFigure 4. Distribution of Mean Sleep Durations for Reliably Symptomatic and Asymptomatic Participants at 4-Weeks Postconcussion eTable 4. Contrast Results for the Exploratory Analyses With the Cognition and Somatic Symptom Subscales [file jamanetwopen-e2516333-s001.pdf]

Supplemental Online Content

Butterfield L, Zemek R, Borghese MM, et al; for the PERC PedCARE team. Nightly sleep duration and symptom burden over one month following pediatric concussion. *JAMA Netw Open.* 2025;8(6):e2516333. doi:10.1001/jamanetworkopen.2025.16333

eMethods.

eFigure 1. Participation Flowchart for the Main Analysis and Sensitivity Analyses

eTable 1. Percentiles of Mean Sleep Duration Over the First Week and First Two Weeks of Recovery

eFigure 2. LOESS Curves of Observed Health and Behaviour Inventory (HBI) vs Mean Sleep Duration at 1-, 2-, and 4-Weeks Indicate Nonlinearity

eTable 2. Contrast Results for the Sensitivity Analysis 1 With Confirmed Sleep Data

eTable 3. Contrast Results for the Sensitivity Analysis 2 With Additional Time-Varying cMVPA Variable

eFigure 3. Distribution of Mean Sleep Durations for Reliably Symptomatic and Asymptomatic Participants at 2-Weeks Postconcussion

eFigure 4. Distribution of Mean Sleep Durations for Reliably Symptomatic and Asymptomatic Participants at 4-Weeks Postconcussion

eTable 4. Contrast Results for the Exploratory Analyses With the Cognition and Somatic Symptom Subscales

This supplemental material has been provided by the authors to give readers additional information about their work.

© 2025 Butterfield L et al. *JAMA Network Open*.

eMethods

An Actical® accelerometer (Model Z, Philips Respironics, Bend, Oregon, USA) was used to monitor activity counts, 24 hours a day for 14 consecutive days, beginning at midnight following enrollment. The participants were instructed to wear the Actical around the waist on the right midaxillary line, and to only remove the Actical when bathing/showering or participating in aquatic activities. The Actical data yielded Actograms, which are an activity count vs time graph developed using Actical® software Version 3.10.0001.

Two raters completed visual inspection of Actograms. Originally, all participants were rated by Rater A. In the second step, Rater B identified issues relating to dates of triage and logbook submissions. Rater B flagged all participants that had evidence of reporting their sleep values on the incorrect day, and/or completed the sleep logs retrospectively. Rater B then re-confirmed Rater A's initial visual inspection of Actograms with correctly dated logbooks to identify those that were submitted incorrectly and those that were submitted retrospectively. An inter-rater reliability analysis was performed. A two-way random effects intraclass correlation coefficient with an absolute agreement definition [ICC(2,k)] found 98.2% inter-rater reliability in mean sleep duration values per week in a subsample of n=96/456 participants. The inter-rater reliability analysis was performed with IBM SPSS Statistics for Macintosh Version 28.0.1.1 (Armonk, NY: IBM Corp.).

Pre-injury mental health diagnostic history was a concatenated binary variable (0=No, 1=Yes) representing diagnostic history of the following conditions: depression, anxiety, sleep disorders, other psychiatric disorder, learning disabilities, attention disorder (ADHD), and other developmental disorder.

© 2025 Butterfield L et al. *JAMA Network Open*.

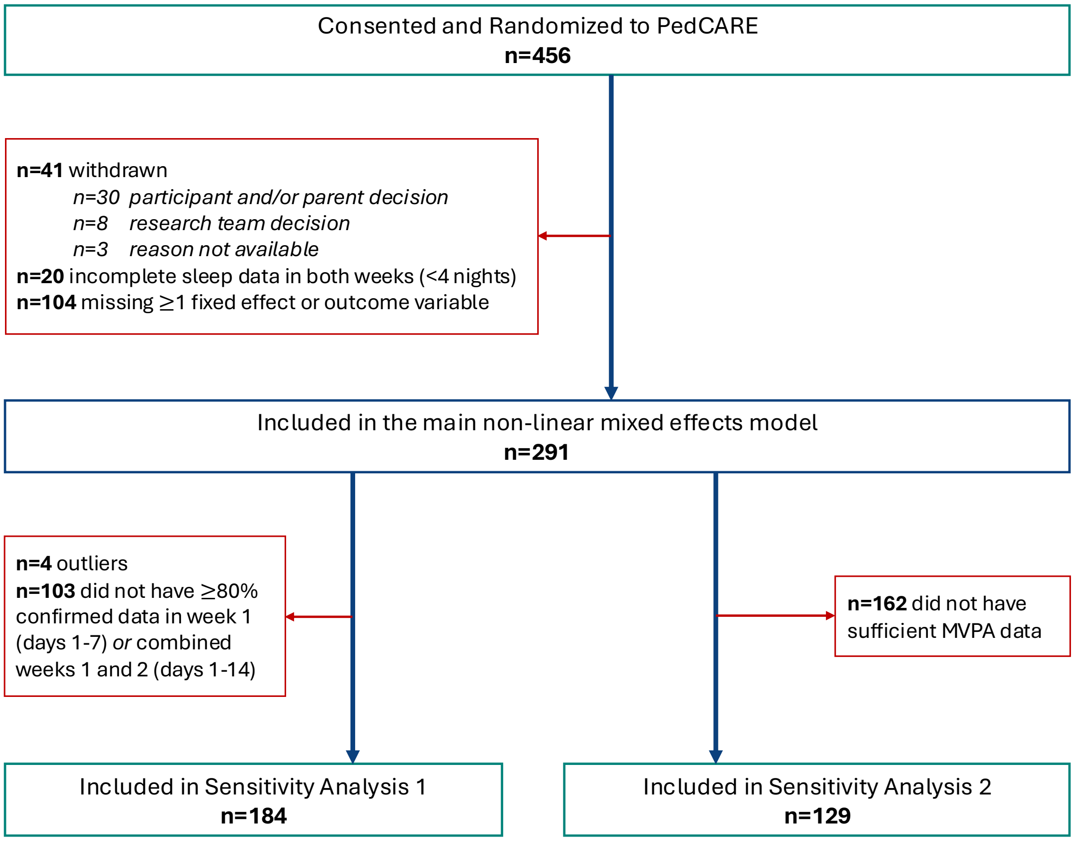

eFigure 1. Participation flowchart for the main analysis and sensitivity analyses.
Confirmed sleep data is defined as data that includes a sleep log and an accelerometer that was worn overnight. MVPA = Moderate-to-Vigorous Physical Activity

© 2025 Butterfield L et al. *JAMA Network Open*.

eTable 1. Percentiles of mean sleep duration over the first week and the first two weeks of recovery.

			Percentile of Mean Sleep Duration (hours)				
**Analysis**	**N**	**Week**	**10th**	**25th**	**50th**	**75th**	**90th**
**Main Mixed Effects Model**	**291**	Nights 1-7	8.8	9.5	10.0	10.6	11.3
		Nights 1-14	8.7	9.3	9.9	10.3	10.9
**Sensitivity Analysis 1**	**184**	Nights 1-7	8.9	9.5	10.0	10.5	11.2
		Nights 1-14	8.8	9.3	9.9	10.3	11.0
**Sensitivity Analysis 2**	**129**	Nights 1-7	8.9	9.5	9.9	10.4	10.7
		Nights 1-14	8.9	9.3	9.7	10.1	10.5
**Exploratory Cognitive Analysis**	**290**	Nights 1-7	8.8	9.5	10.0	10.6	11.3
		Nights 1-14	8.7	9.3	9.9	10.4	10.9
**Exploratory Somatic Analysis**	**290**	Nights 1-7	8.8	9.5	10.0	10.6	11.3
		Nights 1-14	8.7	9.3	9.9	10.3	10.9
**Week 2 Logistic Regression**	**286**	Nights 1-7	8.8	9.5	10.0	10.6	11.3
		Nights 1-14	8.7	9.3	9.9	10.4	10.9
**Week 4 Logistic Regression**	**221**	Nights 1-7	8.8	9.5	10.0	10.6	11.2
		Nights 1-14	8.8	9.3	9.9	10.3	10.9

© 2025 Butterfield L et al. *JAMA Network Open*.

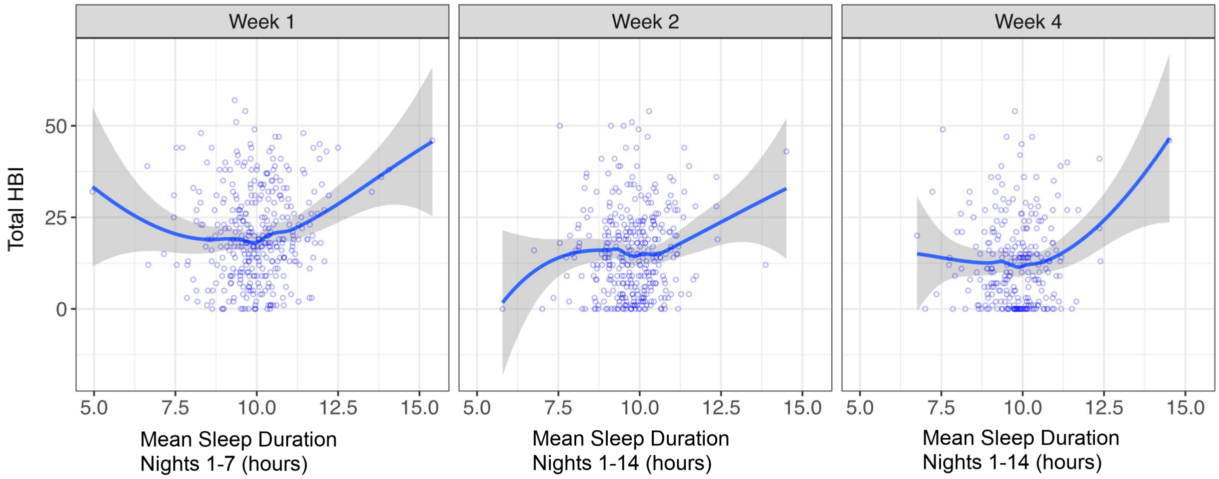

eFigure 2. LOESS curves of observed Health and Behaviour Inventory (HBI) vs mean sleep duration at 1-, 2-, and 4-weeks indicate non-linearity.

© 2025 Butterfield L et al. *JAMA Network Open*.

eTable 2. Contrast results for the sensitivity analysis 1 with confirmed sleep data (n=184)

Week	Contrast (Percentiles)	Estimate[a]	95% CI	SE	Sig., p
1	10th (8.9h) vs 50th (10.0h)	0.5	[-1.71, 2.63]	1.1	0.68
	25th (9.5h) vs 75th (10.5h)	1.5	[0.08, 2.94]	0.7	**0.04***
	50th (10.0h) vs 90th (11.2h)	2.9	[0.46, 5.42]	1.3	**0.02***
2	10th (8.8h) vs 50th (9.9h)	0.2	[-1.99, 2.46]	1.1	0.84
	25th (9.3h) vs 75th (10.3h)	1.1	[-0.51, 2.71]	0.8	0.18
	50th (9.9h) vs 90th (11.0h)	2.4	[0.41, 4.34]	1.0	**0.02***
4	10th (8.8h) vs 50th (9.9h)	0.2	[-1.99, 2.46]	1.1	0.84
	25th (9.3h) vs 75th (10.3h)	1.1	[-0.51, 2.71]	0.8	0.18
	50th (9.9h) vs 90th (11.0h)	2.4	[0.41, 4.34]	1.0	**0.02***

***p<0.001, **p<0.01, *p<0.05, #p<0.1

[a]Estimate of HBI

© 2025 Butterfield L et al. *JAMA Network Open*.

eTable 3. Contrast results for the sensitivity analysis 2 with additional time-varying cMVPA variable

Week	Contrast (Percentiles)	Estimate[a]	95% CI	SE	Sig., p
**1**	10th (8.9h) vs 50th (9.9h)	0.6	[-2.15, 3.25]	1.4	0.69
	25th (9.5h) vs 75th (10.4h)	2.2	[0.31, 4.08]	1.0	**0.02***
	50th (9.9h) vs 90th (10.7h)	3.1	[0.55, 5.61]	1.3	**0.02***
**2**	10th (8.9h) vs 50th (9.7h)	0.2	[-2.27, 2.72]	1.3	0.86
	25th (9.3h) vs 75th (10.1h)	1.4	[-0.25, 3.03]	0.8	0.10#
	50th (9.7h) vs 90th (10.5h)	2.3	[0.42, 4.09]	0.9	**0.02***
**4**	10th (8.9h) vs 50th (9.7h)	0.2	[-2.27, 2.72]	1.3	0.86
	25th (9.3h) vs 75th (10.1h)	1.4	[-0.25, 3.03]	0.8	0.10#
	50th (9.7h) vs 90th (10.5h)	2.3	[0.42, 4.09]	0.9	**0.02***

***p<0.001, **p<0.01, *p<0.05, #p<0.1
[a]Estimate of HBI

© 2025 Butterfield L et al. *JAMA Network Open*.

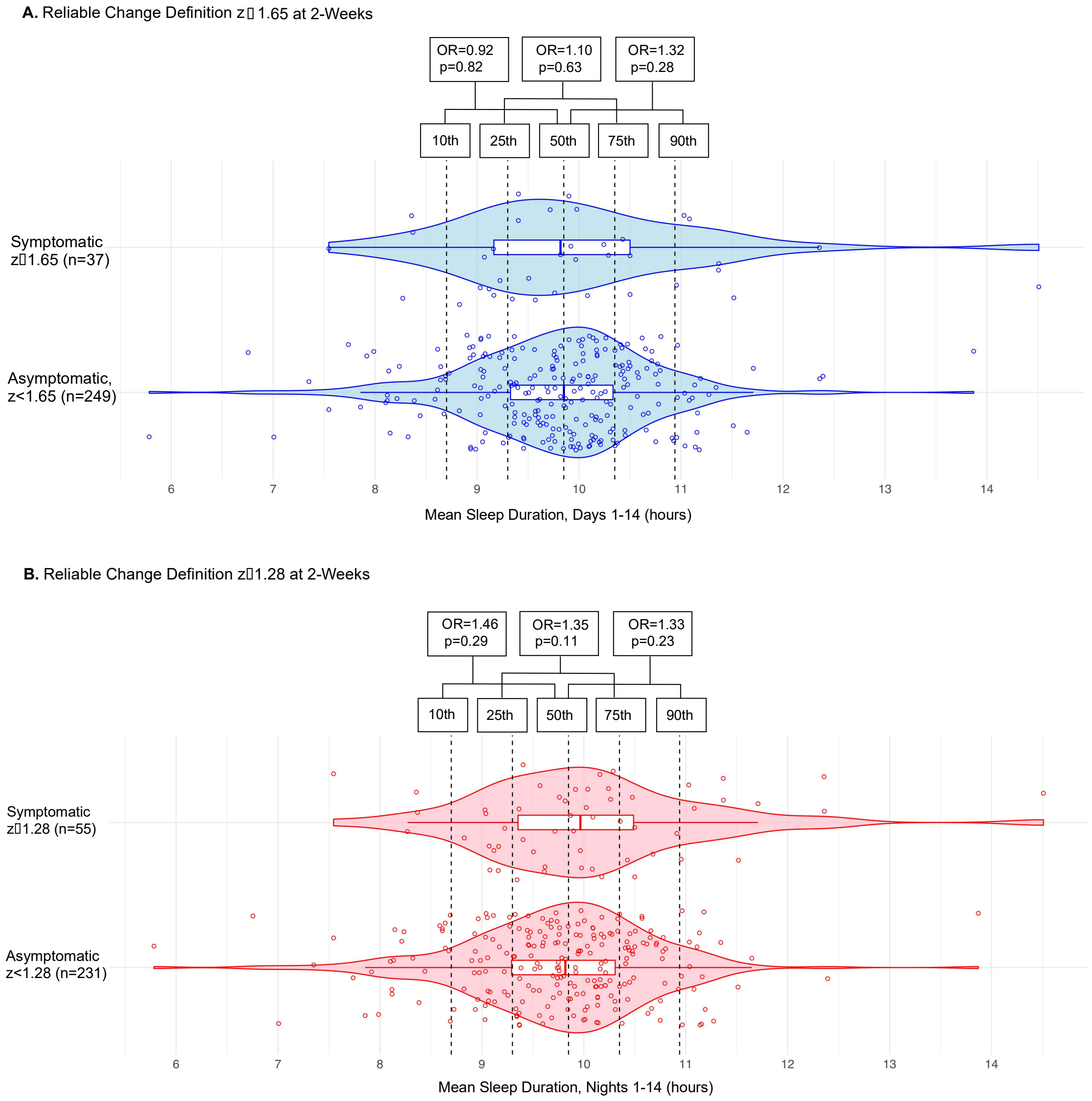

eFigure 3. Distribution of mean sleep durations for reliably symptomatic and asymptomatic participants at 2-weeks post-concussion. A) n=37/286 participants were reliably symptomatic with the conservative definition (z≥1.65). B) n=55/286 participants were reliably symptomatic with the liberal definition (z≥1.28). Vertical lines indicate 10th, 25th, 50th, 75th, and 90th percentiles (left to right). Odds ratios are presented for each of the three contrasts: 10th vs 25th percentiles, 25th vs 75th percentiles, and 50th vs 90th percentiles. PSAC=Persisting Symptoms After Concussion

© 2025 Butterfield L et al. *JAMA Network Open*.

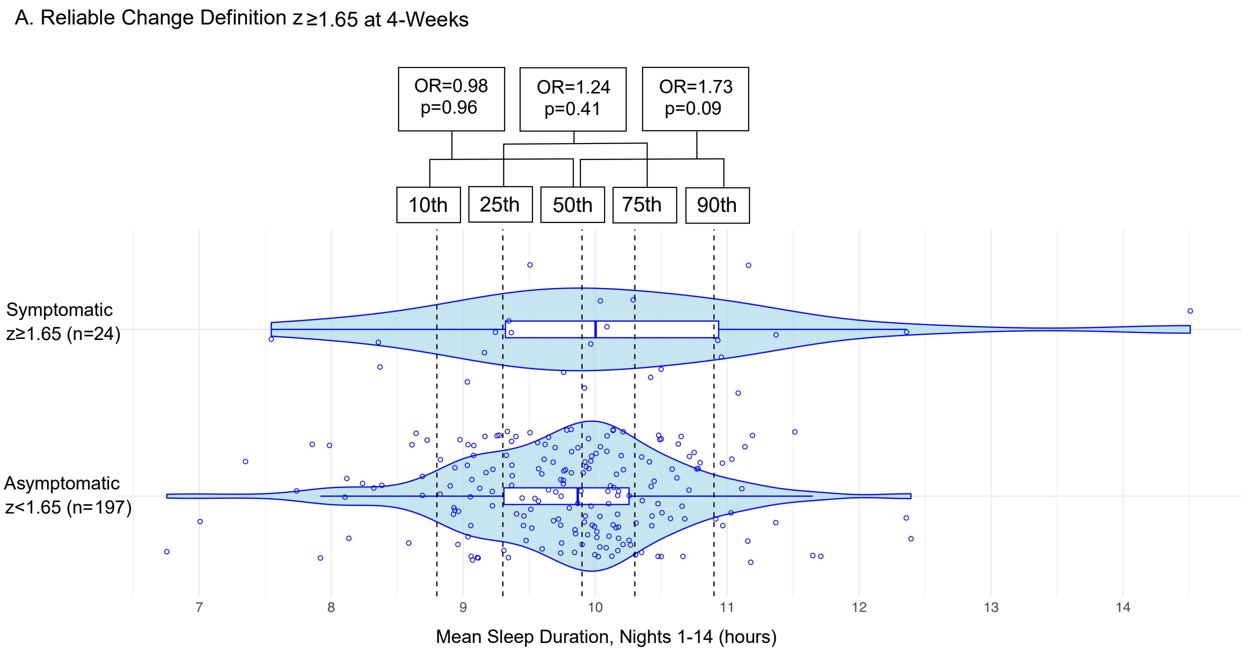

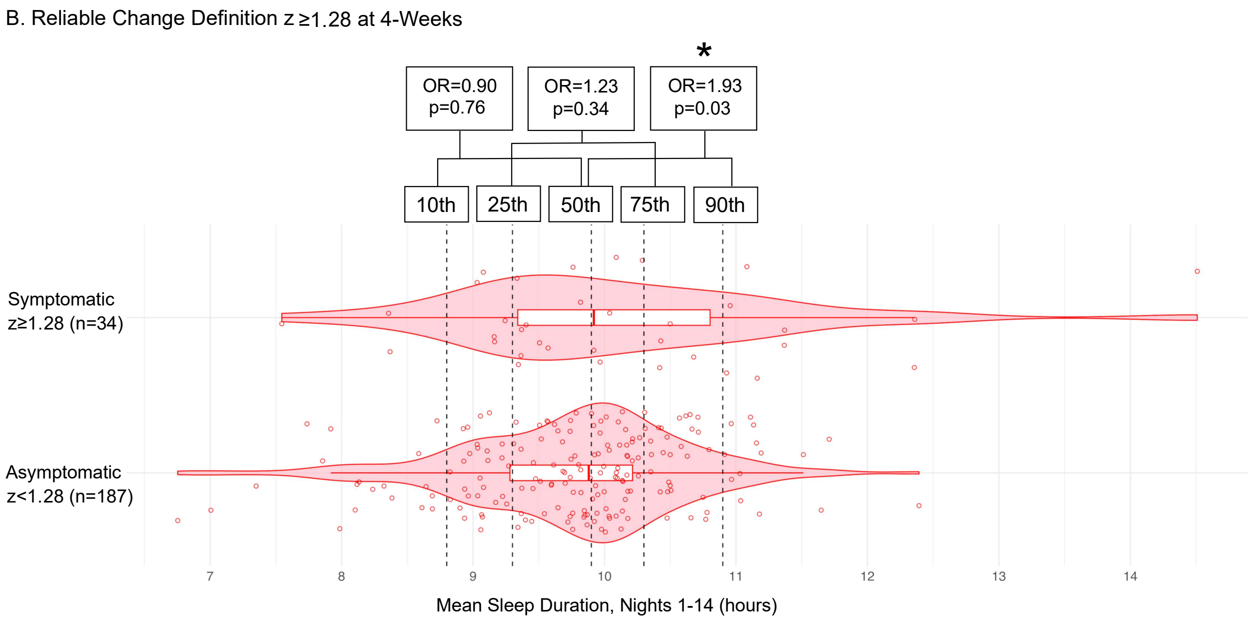

eFigure 4. Distribution of mean sleep durations for reliably symptomatic and asymptomatic participants at 4-weeks post-concussion. A) n=24/221 participants were reliably symptomatic with the conservative definition (z≥1.65). B) n=34/221 participants were reliably symptomatic with the liberal definition (z≥1.28). Vertical lines indicate 10th, 25th, 50th, 75th, and 90th percentiles (left to right). Odds ratios are presented for each of the three contrasts: 10th vs 25th percentiles, 25th vs 75th percentiles, and 50th vs 90th percentiles. Star (*) indicates significant odds ratio ($p<0.05$). PSAC=Persisting Symptoms After Concussion

© 2025 Butterfield L et al. *JAMA Network Open*.

**eTable 4. Contrast results for the exploratory analyses with the cognition and somatic symptom subscales**						
**Subscale**	**Week**	**Contrast (Percentiles)**	**Estimate[a]**	**95% CI**	**SE**	**Sig., p**
Cognition	1	10th (8.8h) vs 50th (10.0h)	-0.04	[-1.05, 0.97]	0.5	0.94
		25th (9.5h) vs 75th (10.6h)	0.6	[-0.02, 1.27]	0.3	**0.06#**
		50th (10.0h) vs 90th (11.3h)	1.3	[0.23, 2.44]	0.6	**0.02***
	2	10th (8.7h) vs 50th (9.9h)	-0.2	[-1.20, 0.90]	0.5	0.78
		25th (9.3h) vs 75th (10.3h)	0.4	[-0.25, 1.00]	0.3	0.24
		50th (9.9h) vs 90th (10.9h)	1.0	[0.16, 1.82]	0.4	**0.02***
	4	10th (8.8h) vs 50th (10.0h)	-0.2	[-1.20, 0.90]	0.5	0.78
		25th (9.3h) vs 75th (10.3h)	0.4	[-0.25, 1.00]	0.3	0.24
		50th (9.9h) vs 90th (10.9h)	1.0	[0.16, 1.82]	0.4	**0.02***
Somatic	1	10th (8.8h) vs 50th (10.0h)	-0.2	[-0.89, 0.41]	0.3	0.47
		25th (9.5h) vs 75th (10.6h)	0.6	[0.22, 1.06]	0.2	**0.003****
		50th (10.0h) vs 90th (11.3h)	1.5	[0.80, 2.26]	0.4	**<0.001*****
	2	10th (8.7h) vs 50th (9.9h)	-0.4	[-1.05, 0.31]	0.4	0.28
		25th (9.3h) vs 75th (10.3h)	0.3	[-0.08, 0.73]	0.2	0.12
		50th (9.9h) vs 90th (10.9h)	1.1	[0.56, 1.66]	0.3	**<0.001*****
	4	10th (8.8h) vs 50th (10.0h)	-0.4	[-1.05, 0.31]	0.4	0.28
		25th (9.3h) vs 75th (10.3h)	0.3	[-0.08, 0.73]	0.2	0.12
		50th (9.9h) vs 90th (10.9h)	1.1	[0.56, 1.66]	0.3	**<0.001*****

***p<0.001, **p<0.01, *p<0.05, #p<0.1

[a]Estimates of HBI subscores

© 2025 Butterfield L et al. *JAMA Network Open*.
