# Supplementary material for: Nightly Sleep Duration and Symptom Burden Over 1 Month Following Pediatric Concussion
Source: JAMA Netw Open. 2025 Jun 18;8(6):e2516333. doi: 10.1001/jamanetworkopen.2025.16333 (PMC12177668; doi:10.1001/jamanetworkopen.2025.16333)
Supplement: Supplement 2. — Nonauthor Collaborators [file jamanetwopen-e2516333-s002.pdf]

Data Sharing Statement

Butterfield. Nightly Sleep Duration and Symptom Burden Over 1 Month Following Pediatric Concussion. *JAMA Netw Open*. Published June 18, 2025.
doi:10.1001/jamanetworkopen.2025.16333

Data
Data available: No

Additional Information
Explanation for why data not available: The data that support the findings of this study are available on reasonable request from the corresponding author with accordance to ethical and legal regulations regarding the sharing of information as to prevent compromising the privacy of research participants.
