# Supplementary material for: Nightly Sleep Duration and Symptom Burden Over 1 Month Following Pediatric Concussion
Source: JAMA Netw Open. 2025 Jun 18;8(6):e2516333. doi: 10.1001/jamanetworkopen.2025.16333 (PMC12177668; doi:10.1001/jamanetworkopen.2025.16333)
Supplement: Supplement 3. — Data Sharing Statement [file jamanetwopen-e2516333-s003.pdf]

Supplemental Online Content: Nonauthor Collaborators
*First name, last name, and suffix (if applicable) are required and will appear in PubMed.

***Group Name(s): PERC PedCARE**

***First Name and Middle Initial(s)**	***Last Name**	***Suffix (eg, Jr, III)**	Academic Degrees	Institution	Location (city, state/province, country)	Role or Contribution, eg, chair, principal investigator	Group (if more than 1 Group listed in the byline) and/or Subgroup (eg, Steering Committee)
Candice	McGahern						
Tyrus	Crawford						
Lauren	Dawson						
Katie	Healey						
Sarah	Reid						
Ken	Farion						
Andrée-Anne	Ledoux						
Roger	Zemek						
Gurinder	Sangha						
Adrienne	Davis						
Nicolas	Reed						
Carol	DeMatteo						
Keith O	Yeates						
Mark	Tremblay						
Michael	Borghese						
